# Implications and Applications of Stretch-Mediated Hypertrophy in Therapy, Rehabilitation and Athletic Training—An Outlook to Future Potential Applications

**DOI:** 10.1007/s40279-025-02237-y

**Published:** 2025-06-06

**Authors:** Konstantin Warneke, José Afonso, Ewan Thomas, Jörn Rittweger, Andreas Konrad, Othmar Moser, Lars H. Lohmann, Astrid Zech, Brad J. Schoenfeld, David G. Behm

**Affiliations:** 1 Institute of Human Movement Science, Sport and Health, University of Graz, 8010 Graz, Austria; 2 Department of Human Motion Science and Exercise Physiology, Friedrich Schiller University, 07749 Jena, Germany; 3 Centre of Research, Education, Innovation and Intervention in Sport (CIFI2D), Faculty of Sport, University of Porto, Porto, Portugal; 4 Sport and Exercise Sciences Research Unit, Department of Psychology, Educational Science and Human Movement, University of Palermo, Palermo, Italy; 5 Institute of Aerospace Medicine, German Aerospace Center (DLR), Cologne, Germany; 6 Department of Pediatrics and Adolescence Medicine, University Hospital Cologne, Cologne, Germany; 7 Division of Exercise Physiology and Metabolism, Department of Sport Science, University of Bayreuth, 95440 Bayreuth, Germany; 8 Interdisciplinary Metabolic Medicine, Division of Endocrinology and Diabetology, Department of Internal Medicine, Medical University of Graz, 8036 Graz, Austria; 9 Department of Human Motion Science and Exercise Physiology, University of Hamburg, 20148 Hamburg, Germany; 10 Department of Exercise Science and Recreation, CUNY Lehman College, Bronx, NY USA; 11 School of Human Kinetics and Recreation, Memorial University of Newfoundland, St. Johns, NL A1C 5S7 Canada

## Abstract

Muscle strength and hypertrophy are of high importance for almost every sport, but also for more general prevention and therapeutical approaches. While the most common way to enhance functional and structural muscle capacities is resistance training, there are scenarios in which a resistance training routine may not be feasible or may even be contraindicated. Recently published works showed the potential of high-volume static stretching programs when it comes to promoting muscle strength and hypertrophy, albeit with comparatively long stretching durations per bout in comparison with resistance training. Therefore, there is limited practical applicability of this training approach for healthy participants with access to dynamic training facilities and supervised training. However, there are potential settings in which stretch-mediated hypertrophy could be useful and should be investigated. This current opinion paper explores such potential settings, including in immobilization-induced atrophy, in patients with type 2 diabetes, and as a supplement to common resistance training routines to increase the accumulated volume of mechanical overload of the muscle in healthy or athletic populations. Static stretching might also be used to counteract atrophy in spaceflight because other forms of training that may induce sufficient levels of mechanical strain seem infeasible or impractical. Thus, we explore the potential applications of static stretching routines while considering the feasibility and opportunity for their practical implementation. Consequently, this current opinion paper provides a demand for further investigations of static stretch-mediated adaptations as a potential passive alternative with a focus on therapy and prevention.

## Key Points

**Table:** 

While stretching is commonly associated with flexibility increases, recent evidence proposed stretch-mediated hypertrophy and therefore a new avenue in practice and rehabilitation.
Although for healthy participants unpractically high volumes are necessary to reach effects known from lower dosages in resistance training, there is promising potential in prevention, rehabilitation and therapy for individuals and patients with restricted access to common treatment options.
This work outlines these potentials and derives further experimental studies and clinical trials to explore the applicability in these settings.

## Introduction

While stretching is mostly performed to improve range of motion, there may be applications that receive less attention and deserve further consideration [1]. A promising avenue of research is the application of static stretching to promote stretch-mediated muscle hypertrophy and strength increases [2, 3], which can promote benefits for health, therapy and rehabilitation.

Mechanical overload is paramount for muscle hypertrophy and strength improvements [4]. This stimulus is commonly induced using external loads via resistance training, initiating a cascade of anabolic transcriptional factors [e.g. mechanistic target of rapamycin (mTOR)/ribosomal protein S6 kinase 1 (P70S6K)/phosphoinositide 3-kinase (PI3K)] that ultimately leads to an increased muscle protein synthesis rate [5–7]. Muscle size and maximal strength are not linearly associated, but larger muscle cross-sectional area correlates with an increased potential to exert higher muscle forces [8]. As indicated, mechanical overload can be alternatively induced via stretching, which means static stretching is a tool used to induce (high-magnitude and long-duration) passive tension to overload the muscle(–tendon unit) and hence elicit adaptations in muscle strength and size. When performed with sufficient volume, animal and in vitro studies have reported large increases in muscle size [9, 10] and strength [11] using stretching protocols of 30 min to 24 h per day over several weeks. While 24 h could be considered impractical in humans, Frankeny et al. [12] proposed that 30 min of daily stretching “was within normal physiological limits” (pp. 275–276). The authors suggested that these routines could be a viable alternative or supplementation to common exercise routines in special situations, such as counteracting immobilization-induced atrophy [13].

While the first human studies assessing muscular strength and size adaptations with research designs exceeding 60 min of stretching per week were performed in 2021 [14, 15], Warneke et al. [16] were the first to use an orthotic device to stretch the plantar flexors [16, 17] with stretching durations comparable to those in animal research (up to 2 h per day for 6 weeks) and find meaningful effects. While this proof-of-principle research was limited to the plantar flexors, subsequent studies confirmed the applicability to the pectoralis major [18–20] and the rectus femoris [21]. Underlying mechanisms [22, 23] as well as functional (maximal strength) and morphological (muscle thickness) responses [19, 24] were proposed to be similar between active resistance training and passive stretch-induced overload tension [2, 25, 26], although stretching seems less promising under practical time considerations [27]. While strength and hypertrophy showed a similar magnitude [24], stretch training required much higher volumes compared with resistance training in the direct comparison in the plantar flexors (7 × 1 h per week versus 3 × 45 min per week, respectively), making stretching an unreasonable substitution in healthy and active populations [27, 28].

However, several populations encounter different barriers to participate in active, supervised resistance training. In some specific settings overloading muscles to counteract atrophy or induce hypertrophy via active training is contraindicated or inaccessible. These may include, but are not limited to, frail elderly patients or those with pre-existing health conditions such as patients who have osteoarthritis, are bedridden, or have injury-related mobility restrictions via immobilization.

## A Brief Summary of the Current Literature on Stretch-Mediated Hypertrophy and Strength Increases

First evidence for stretch-induced muscle mass and strength increases emerged in animal models in the 1970s [29], with the bulk of the studies published up to the early 2000s [30]. A 2022 systematic review with meta-analysis on hypertrophy in animal models reported stretching protocols ranging from 30 min to a continuous 24 h per day for up to 6 weeks that led to high-magnitude muscle mass increases (*d* = 8.51) [9] with a peak muscle mass increase of ~ 318% within 35 days [31].

Despite the evidence in animals and Frankeny and colleagues’ request to investigate the transferability to humans in 1983 [12], human studies with comparable daily stretch durations, application frequencies and overall volumes only emerged within the past 5 years. In their recent systematic reviews with meta-analyses Arntz et al. [3, 28] and Warneke et al. [2, 22] concluded that chronic static stretching has the potential to positively influence muscle strength and size – especially with a high session duration (> 15 min per muscle) and moderate weekly frequency (> 3 × per week) resulting in a high overall stretching volume for a given muscle compared with previous studies [32]. The highest increases were found in studies by Warneke et al. who applied splint-based plantar flexor stretching for 30 min to 2 h per day every day for 6 weeks [16, 17, 24, 33]. However, stretch-induced adaptations were not only found in the relatively small plantar flexors. While Wohlann et al. [21] reported stretch-induced maximal strength without significant muscle thickness increases for the rectus femoris, Reiner et al. [18] and Wohlann et al. [19, 34] confirmed that stretching can also elicit these adaptations in the pectoralis major.

Additionally, two studies reported stretching with high volume and intensity induced hypertrophy and strength increases similar to a resistance training program. Wohlann et al. [19] found 15 min of pectoralis major stretching four times per week to be as effective as five sets of 10–12 repetitions of dynamic resistance training with a “butterfly/pec deck machine” three times per week as both resulted in about 5–7% muscle thickness and ~ 10% isometric maximal strength increases. Although, Warneke et al. [24] reported slightly superior hypertrophy in the resistance training group (+ 8.5% versus about 5% in gastrocnemius lateralis and about 8% in gastrocnemius medialis), the isometric maximal strength increases with extended knee joint were slightly higher in the stretching group (+ 18% versus + 13.36%), whereas the flexed knee joint condition favoured the resistance training group (+ 9.96% versus 9.58%). Notably, the pre–post training differences were insignificant (*α*-level set to 0.05) for all these parameters.

In a narrative review on the physiology of these stretching effects, Warneke et al. [22] discussed that the underlying mechanisms behind resistance training and stretching adaptations on muscle strength and size might be similar. Although specifics are debated, high mechanical tension on the muscle(–tendon unit) is believed to be the key driver of IG-1 and mTOR/p70S6K signalling, as the proposed mechanism controlling protein synthesis and in turn hypertrophy [4, 35].

Although of lower time-efficiency, we see future potentials that should be investigated to explore whether passive static stretching can be a viable therapeutic substitution or supplementation to optimize current training routines. On the one hand, its easy integration into daily seated activities, such as working on a computer or watching television [36], outline the value for future research to explore applicability in different health- and rehabilitation-related areas ranging from astronauts to clinical settings (e.g. patients who are immobilized). On the other hand, while the required 15 to 60 min of stretch per muscle call the sole application in athletic populations into question, the work will introduce the future potential as a combined approach.

## Counteracting Immobilization-Induced Atrophy

A traumatic injury with or without subsequent surgery can lead to partial immobilization [37]. Even if other body regions are engaged in exercise and benefit from non-local effects [38, 39], regional musculoskeletal atrophy may occur in the non-exercised muscles [40]. Prominent examples include anterior cruciate ligament ruptures and ankle fractures. While the anterior cruciate ligament rupture has a high prevalence amongst younger populations [41] and is quantified with an annual incidence of 30 to 78 per 100,000 persons [42], ankle fractures are more commonly found amongst elderly populations. An analysis of the Swedish Fracture Register during a 10-year period reports the patients’ mean age for ankle fractures to be 55 years, with an overall annual incidence of 152 per 100,000 people [43]. This injury has a high incidence rate, especially among older adults, and leads to severe loss of muscle mass. For example, it has been shown to lead to ~ 14% muscle atrophy in the plantar flexors in just 2 weeks [44].

Restoration of such atrophy relies, for the most part, on post-surgery rehabilitation facilities. These utilize inter alia resistance training, which may restore physical capacity and muscle size while reducing atrophy [45, 46] during the rehabilitation process. Still, participants are regularly left with inter-limb muscular strength imbalances of up to 20% after their program is completed [44]. Since the effectiveness of resistance training in restoring muscle size and strength is well investigated, the lack of success in fully restoring lost functionality could be attributed to a lack of motivation and commitment of the participants [47, 48]. Even among athletes, patients who are recovering may have a limited number of strength training or general physiotherapy sessions per week [49–51], while others fear exercising without supervision may increase the chance of re-injury [52, 53].

In contrast to active training, passive stretching could be considered an effective option or supplement without the necessity of supervision [34] while being considered safe, thereby alleviating/dispelling the fear of re-injury when exercising alone. This practice could be a valuable complement to strength training or other therapies—especially in early rehabilitation stages [22]. Hereby, interventions may be considered successful even without hypertrophy, as the reduction of the rate of atrophy reflects a positive outcome in itself [54].

Due to the aforementioned stretch-mediated hypertrophy effects, higher volumes of static stretching could conceivably prevent or reduce the degree of muscle atrophy and wasting by incorporating stretching exercises at intervals throughout the day and week [55, 56]. In the context of supervised sessions, assisted stretching may be utilized if more active training alternatives such as resistance training are infeasible or even contraindicated [57, 58]. This rationale can be extended to other immobilization conditions, such as prolonged illness or recovery from surgery, during which patients may be bedridden, and therefore static stretching applications could prove useful in reducing muscle wasting through stretch-mediated hypertrophy. It is worth considering the potential apprehensions of patients who are more fragile, who may be reluctant to engage in strength training but more willing to participate in stretching exercises. Assessing such preferences may be crucial, and stretching should be considered as a viable option.

Importantly, the effects of stretch-mediated hypertrophy during early rehabilitation stages are currently speculative. Although Warneke et al. [59] showed that 1 h of daily stretching was sufficient to counteract strength and range-of-motion muscle imbalances between the left and right lower leg, these results came from healthy participants. Research specifically designed to investigate the magnitude and benefits of stretch-mediated hypertrophy or reduced atrophy during immobilization periods (e.g. early post-injury rehabilitation stages) is warranted.

## Potential Contribution to Preventing Falls or Declining Motor Function in Older Adults

The ability to maintain and restore postural control during gait or upright standing is of crucial importance in everyday life. This is especially true for orthopaedic patients and older adults because these populations are prone to severe fall-related injuries [60, 61]. The ongoing demographic changes in many societies pose several challenges. One such challenge pertains to an increased need for medical care and nursing in the future: one-third of people > 65 years and half of people > 80 years of age fall at least once per year [60]—and those societies are continuing to age. Therefore, effective exercise routines to maintain motor function are desperately needed.

Motor function and balance can be considered multifactorial constructs heavily influenced by sensorimotor control [62]. Balance is, however, also positively correlated with muscle strength [63]. This link is not surprising, since sedentary behaviour and reduced physical activity are closely related to lower levels of muscular strength [64] and, in turn, low levels of lower-extremity strength discriminate fallers from non-fallers [65]. Accordingly, the American Geriatrics Society and American Academy of Orthopedic Surgeons panel on fall prevention lists muscular weakness and functional impairments as the most prominent risk factors for falls in elderly patients [66]. While sensorimotor control is frequently triggered when performing a variety of exercises on unstable surfaces [67], the aforementioned American panel [66] emphasized the need for strength-increasing exercise routines. Unsurprisingly, the 2022 “World Guidelines for Falls Prevention and Management for Older Adults: A Global Initiative” strongly recommend utilizing individualized progressive resistance training [68]—which has shown positive effects on balance performance, especially if training was supervised [69].

However, a survey study found that older adults list (a) limited access to facilities and not having enough time as well as (b) being too tired, not knowing how to perform the exercises and being too lazy as external and internal barriers, respectively, for participation in physical activity [70]. Since strength in general [63], and plantar flexors’ muscle strength and size in particular [71, 72], were positively correlated with balance performance, the request to investigate the transferability of stretch-mediated hypertrophy and strength increases seems reasonable as a means of providing a passive and safe exercise alternative which is effective when unsupervised [34]. Additionally, static stretching, e.g., via splint, is more convenient than other forms of resistance exercise, as it can be performed in virtually any location without the need for gym-based equipment. Although a recent meta-analysis on stretch-mediated balance performance shows no clear evidence regarding chronic improvements, the interpretation is limited by short stretch-training durations and low overall stretching dosages in the included studies that must be considered insufficient to model muscle strength and size [73].

While static stretching might therefore still be an effective intervention with enhanced exercise commitment and adherence rates, it must be emphasized that we are not insinuating that stretching is as effective as resistance training in this regard but rather proposing it as an alternative in cases where resistance training is not viable.

## Stretch-Mediated Hypertrophy and Strength Increases in Diabetes

Diabetes is a disease that is characterized by chronic hyperglycaemia and impaired glucose metabolism due to insufficient or missing endogenous insulin production and/or decreased sensitivity to insulin. The largest proportion within this pathology is type 2 diabetes, affecting approximately 14% of the worldwide population [74]. The disease is thus perceived as a global health burden, affecting peoples’ quality of life and functional capacities with significantly increased levels of morbidity and premature mortality while also resulting in tremendous healthcare-related expenditures [75]. In general, physical activity and exercise are recommended in the treatment of people with type 2 diabetes [76]; next to the effects on force development, locomotion and joint stability, skeletal muscles are crucial for metabolic health in people with diabetes [77].

Musculature especially supports the blood glucose equilibrium by means of intramuscular glucose-absorbing efficacy [78]. In previous studies, it was shown that aerobic exercise or resistance training as well as combined approaches have beneficial effects on long-term glycaemia, measured via glycated haemoglobin in adults with type 2 diabetes [79]. While a combined training approach was most beneficial in improving glycaemia, Al-Ozairi et al. [80] called for alternatives to enhance muscle mass as a crucial moderator for metabolic diseases and dysfunctional blood glucose regulation in people with type 2 diabetes. Aerobic exercise and resistance training are advisable for these patients, but they may not have access to equipment and facilities, and pathologically increased body mass makes some exercise routines difficult to perform (e.g. jogging subjects joints to heavy loads). To counteract these barriers to exercise, exercise prescriptions need to be personalized on the basis of the constitution and comorbidities of people with type 2 diabetes [81].

Interestingly, in a recent meta-analysis it was shown that stretching can improve glycaemia in people with type 2 diabetes [82]. This analysis indicated that stretching exercise acutely decreases blood glucose levels and chronically improves glycated haemoglobin levels. Hence, from a clinical perspective, stretching might help to promptly decrease, e.g., post-prandial glucose levels and constantly increase the chance of near-physiological long-term glycaemia. From a physiological point of view, stretching exercise, performed actively or passively, might increase the amount of circulating and cell-surface-docking intramuscular insulin-independent glucose transporter type 4 (GLUT-4) [83]. On a cellular level, stretching might increase the accumulation of AMPK, ROS, NO and CaMK, which are linked to greater TBC1D1 (also known as AS160) phosphorylation on S237 [84] when compared with exogenous insulin-induced GLUT-4 mobility [83]. When insulin is exogenously administered or endogenously produced under post-prandial conditions, TBC1D2 is the main trigger to move GLUT-4 to the cell surface to initiate intracellular glucose uptake [83]. Stretching exercise may be assumed to have a longer and more intense duration of action on cellular glucose uptake than most of the exogenously applied bolus insulins [85].

Since high volumes of stretching were sufficient to meaningfully increase muscle hypertrophy and strength comparable to those of a RT routine [19, 24], it seems reasonable to further explore this training modality as a complement to common routines, or as their replacement in cases where they are not viable. This is mainly based on the fact that high volumes of stretching and regular resistance training increase muscle hypertrophy [19, 24], and increases in muscle mass were shown to improve long-term glycaemia [86].

## Potential Applications in Space

A well-known setting that results in severe muscle loss is space flight, in which the microgravity negatively affects astronauts’ health, inter alia with a rapid decline in muscle size and strength [87]. In space, ground reaction forces are substantially lower than in a comparable Earth setting [88, 89], resulting in a significant unloading of the musculoskeletal system. Thus, unsurprisingly, lower extremity muscles, and especially the plantar flexors, exhibit the bulk of the muscle mass decrease [90, 91]. Research on four male astronauts who spent 181 ± 15 days on the International Space Station reported a decrease in calf muscle volume of between 10 and 16% [90] – despite the use of countermeasure exercises that included treadmill running and cycle ergometry [78, 79]. This highlights that direct research is restricted owing to the limited number of people actually experiencing spaceflight.

Experimental bed rest is an often-used, broader-scale, ground-based model of spaceflight that induces many of the same deconditioning effects of spaceflight, albeit at a somewhat slower pace [92]. In bed rest studies, resistance training emerged as a potential countermeasure of disuse-induced atrophy—which is also already used in spaceflight [93]. However, meaningful resistance training that can positively influence muscle volume and strength in deep space is challenging, as gravity must be replaced by pull-down devices. These devices inflict discomfort and pain, and astronauts therefore perform treadmill running with pull-down forces that are below their body weight [94]. Moreover, there is no body weight in space, so to achieve the same musculoskeletal loading, the substituting pull-down forces would have to be even larger than gravity’s pull on earth. Accordingly, these obstacles limit the applicability of exercise routines to induce sufficient mechanical strain, which was outlined to be of vital importance to maintain contractile and metabolic parameters and therefore muscular health [95].

Since current exercise routines seem to be insufficient to counteract substantial muscle wasting after their return from International Space Station missions [90, 96, 97] interventions that effectively induce a continuous mechanical overload were outlined to prevent a further decline of contractile and morphological parameters [95]. As early as 2001, Yamashita-Goto et al. [98] referred to stretching as an effective countermeasure to prevent muscular maladaptation due to bed-rest-based unloading. It seems surprising that no studies have included high-volume stretching interventions with sufficient stretching durations to induce muscle hypertrophy in bed rest studies within the last > 20 years.

In summary, given the physical difficulty in performing earth-like countermeasure exercise in microgravity, passive or semi-passive stretching exercise may be an intriguing addition, with the potential to enhance muscle mass on earth and attenuate muscle atrophy in space. However, no direct evidence of stretch-mediated hypertrophy in space or microgravity currently exists in humans.

## Relevance of Stretch-Mediated Hypertrophy for Athletes

To date, there is limited evidence on stretch-mediated hypertrophy in athletes. Just one randomized controlled trial [33] on athletes and one case study [99] on a competitive, elite-level, drug-free bodybuilder (~ 20 years of resistance training experience) found stretch-mediated hypertrophy in resistance-trained populations. The reason for the limited evidence might be due to impractically long stretching times of 1 h of daily stretching for a single muscle group to elicit strength and hypertrophy, whereas comparable effects can be achieved with common dynamic resistance training routines in a fraction of that time [27]. Although shorter-duration stretching routines showed small, albeit statistically significant strength increases [21, 100], these protocols were not accompanied by muscle hypertrophy, which required longer stretching durations (≥ 15 min) [2]. Such small stretching durations were also not sufficient to enhance speed and athletic performance. While earlier studies indicated potential benefits of stretching for running and sprinting [101, 102], emerging evidence refutes these findings. For example, Arntz et al. [3] did not detect chronic stretching benefits on explosive strength. Moreover, Warneke et al. [103] found no evidence that chronic stretching programs sufficiently improved jump or sprint performance.

While the substitution of static stretching for active resistance training in healthy participants or athletes seems unreasonable, future studies should evaluate supplementary effects. The aforementioned case study by Homer et al. [99] on a competitive bodybuilder illustrates that high-volume (6 × per week for 1 h per session), high-intensity (8/10 pain level) static stretching in addition to high-volume (20 sets of straight knee calf raises per week), high-intensity (between two repetitions in reserve to momentary failure) habitual resistance training can induce meaningful gastrocnemius muscle thickness increases (up to 23.4%) within 12 weeks. The authors of the study concluded that “any measurable hypertrophy occurring over a relatively short period in a highly trained athlete is arguably notable” ([99], p. 5). It should be noted that this was a case study on a single individual and on an isolated, small muscle group; thus, caution is warranted when drawing inferences, and further investigation using randomized controlled designs is needed to better understand the associated adaptations of the strategy.

Also, the combination of both resistance training and high-intensity inter-set stretching could be an interesting approach [27] to maximize mechanical tension with the potential to save time or optimize the effectivity of dynamic training routines [22, 104] by supplementing passive overload [14, 103]. The benefits of this approach remain hypothetical, and further research is needed to elucidate the topic.

## What Dosage is Needed for Stretch-Mediated Hypertrophy to Occur?

As with any intervention, dosage should be carefully considered, particularly during the early stages of rehabilitation. High-volume stretching of > 15 min per muscle group seems to be required to promote muscle hypertrophy [2], but this is based on a handful of studies, most of them performed with untrained individuals, and none of the high-volume studies lasted more than 8 weeks, so there is no evidence on longer-term interventional effects (except a case study performed by Homer et al. [99]). Additionally, it remains unknown how such volumes are optimally distributed to induce maximal effects. Therefore, it is unclear whether 15 min should be split into, for instance, 3 × 5 min or whether it is necessary to perform a continuous 15-min stretch; further research is needed for clarification. Also, the role of intensity in stretch-mediated hypertrophy is largely unexplored [22]. While, for other exercise modalities (resistance and aerobic training), intensity may be calculated as a percentage of a maximal load or a percentage of maximal heart rate (respectively), the intensity of stretching is determined as a subjective function of personal discomfort or pain. Therefore, this may vary not only across individuals but also within individuals according to several conditions (i.e. time of the day, stress, hydration, mood, etc.). This poses further difficulties in the comprehension of mechanisms related to stretch-mediated hypertrophy. A balance must be struck between the intensity required to promote some degree of hypertrophy or diminished atrophy while ensuring the safety of the affected region. Another trade-off is whether the intensity needed to promote muscle hypertrophy or reduce atrophy coincides with the intensity needed to elicit stretch-mediated pain reduction [105]. For example, stretching-induced range of motion improvements in healthy subjects seem to benefit from intensities reaching the pain threshold [106, 107]. Research with populations undergoing early rehabilitation stages must ascertain what the minimum necessary intensity to promote stretch-mediated hypertrophy or reduced atrophy is while staying within acceptable limits of perceived pain and ensuring compliance with the interventions.

## Conclusions and Outlook

**Fig. 1:**
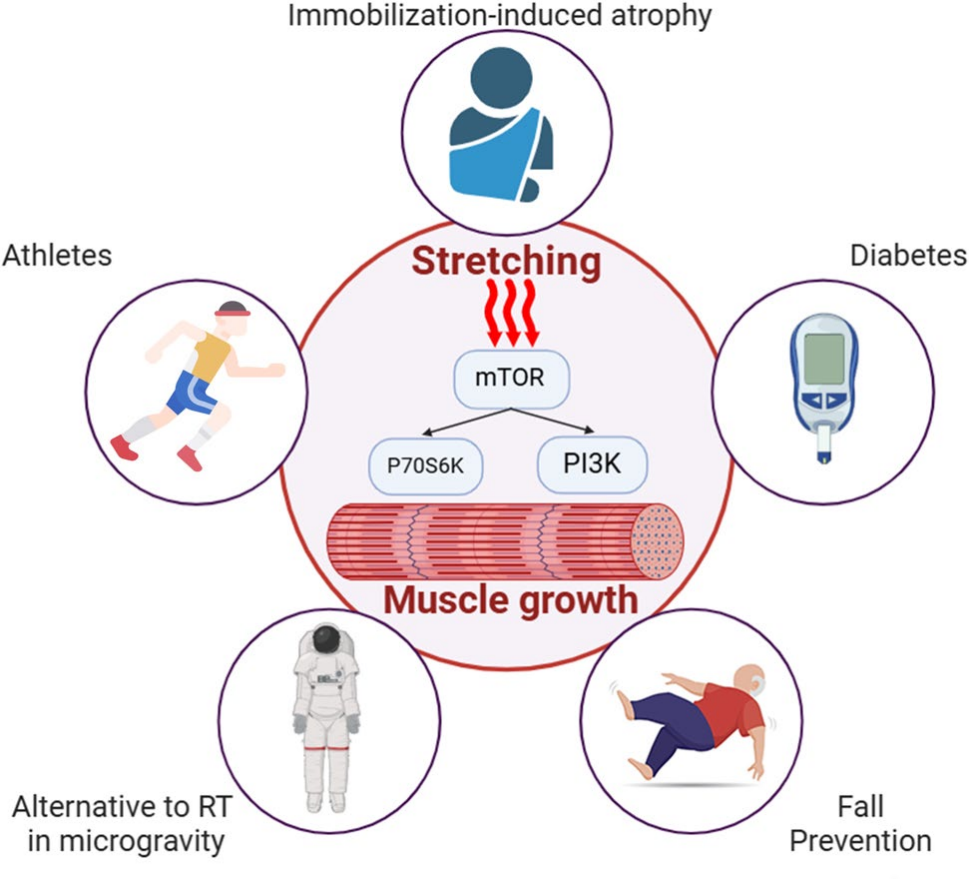
Graphical summary of outlined applications. *mTOR* mechanistic target of rapamycin, *P70S6K* ribosomal protein S6 kinase 1, *PI3K* phosphoinositide 3-kinase, *RT* resistance training

In this current opinion paper, we highlighted future potential applications, for example, with elderly patients, in whom greater muscle mass is positively associated with increased longevity, while greater strength and muscle compliance can improve balance, contributing to reductions in trips and falls, respectively. However, the practical implications to improve muscle strength and hypertrophy with stretching seems limited for healthy and active participants. Nonetheless, stretching could provide several benefits for individuals with restricted access or individuals for whom active training is contraindicated. Chronic stretch training may also play a role in metabolic functioning, such as glucose sensitivity with diabetes. While in astronauts, high volumes of stretching could help to prevent muscle atrophy and strength loss, athletes, especially highly resistance-trained individuals, might benefit from supplementary static stretching interventions as a means to further enhance mechanical overload, thus maximizing hypertrophy effects (Fig. 1). However, the practical relevance in people with access to common resistance training programs seems negligible. Nevertheless, much effort must be invested in future research, as for most topics, clear and experimental evidence is lacking.

## Declarations

### Data availability statement

N/A.

### Code Availability

N/A.
